# Effect of decreasing population growth-rate on deforestation and population sustainability

**DOI:** 10.1080/19420889.2021.2010394

**Published:** 2021-12-18

**Authors:** Gerardo Aquino, Mauro Bologna

**Affiliations:** a Goldsmiths, University of London, London, UK; b Departamento de Ingeniería Eléctrica-Electrónica, Universidad de Tarapacá, Arica, Chile

## Abstract

We consider the effect of non-constant parameters on the human-forest interaction logistic model coupled with human technological growth introduced in [1]. In recent years in fact, a decrease in human population growth rate has emerged which can be measured to about 1.7% drop per year since 1960 value, which coincides with latest UN projections for next decades up to year 2100 [2]. We therefore consider here the effect of decreasing human population growth-rate on the aforementioned model and we evaluate its effect on the probability of survival of human civilization without going through a catastrophic population collapse. We find that for realistic values of the human population carrying capacity of the earth (measured by the parameter β) this decrease would not affect previous results, leading to a low probability of avoiding a catastrophic collapse. For larger more optimistic values of β instead, a decrease in growth-rate would tilt the probability in favor of a positive outcome, i.e. from 10–20% up to even 95% likelihood of avoiding collapse.

## Introduction

The problem of the survival of humanity, for long time the subject of science fiction and catastrophist movies, has recently become central in both scientific and social debate, due to various factors, among them climate changes, intensive exploitation of resources and more generally a deterioration of the planetary ecosystem. Recently, the authors of Ref [1] pointed out the serious repercussions on the life of the planet of uncontrolled deforestation. The model examined in [1] considered the strong connection between the use of the resources (i.e. the forests) and the technological development [1,2] governed by the following equations

(1) $$\frac{d}{dt}N\left(t\right)=rN\left(t\right)\left[1-\frac{N\left(t\right)}{\beta R\left(t\right)}\right],$$

(2) $$\frac{d}{dt}R\left(t\right)=r^{\prime }R\left(t\right)\left[1-\frac{R\left(t\right)}{R_{c}\left(t\right)}\right]-a_{0}N\left(t\right)R\left(t\right)$$

for the the world population *N* and the forest-covered surface *R*. The parameters involved in Eqs. (1) and (2) are: *β*, a positive constant related to the human population carrying capacity of the earth, *r* the growing rate for humans (estimated as *r* ~ 0.01 yr^−1^) [3], *a0* which may be identified as the technological parameter representing the ability of exploiting the resources, $r^{\prime }$ the renewability parameter characterizing how quickly the resources are able to regenerate and finally *Rc*, the resource carrying capacity of the earth that in our case may be identified with the initial 60 million square Kilometers of forest.

(3) $$\frac{1}{R}\frac{dR}{dt}\approx -a_{0}N$$

The actual population of the earth is *N* ~ 7.5 × 10^9^ inhabitants with a maximum carrying capacity estimated [4] of *Nc* ~ 10^10^ inhabitants. The forest carrying capacity may be taken *Rc* ~ 6 × 10^7^ Km^2^ of forest [5] while the actual surface of forest is R∼ x<4×107 Km^2^. We may estimate the *β* parameter as *β* ~ *Nc/Rc* ~ 170 or using actual data of population growth [6]. In this case we obtain *β* ~ 700. These estimations have a certain degree of ambiguity. The approach to estimate *β* = 170 has been used in Ref [2]. and gives results in good agreement with the archeological data. But, differently of our case, i.e., the Earth, for the maximum carrying capacity of Easter Island, it was relatively easy to get an accurate estimation. The second range of β estimations based on the actual data of growing population, β ~ 700, even if in principle could be accurate, it suffers from a limited window of time and could be affected by population fluctuations. For these reasons, we consider a range 170 ≤ β ≤ 700 in the numerical simulations.

The system (1)-(2) is complemented by the equation describing the technological development

(4) $$\frac{d}{dt}T=\alpha T\xi \left(t\right).$$

where *T* is the technological level that we identify with the energy consumption, *α* a constant parameter describing the technological growth and we may, rather optimistically, choose the value *α* = 0.345 yr^−1^ following the Moore Law [7,8]. Finally, *ξ(t)* is a random variable with values 0, 1. The time durations of the states 0, 1 are characterized by a waiting time distribution density *ψ(t)*. The random variable takes in account the fact that the technological development can have stops due to the economic investments on the spatial technology.

The above model, Ref [1]., assumes that the parameters *r, β*, $r^{\prime }$, *Rc, a0, α* have constant values over time. Instead, in this short communication we focus on the time dependence of the parameter *r* that is directly related to the reproductive capability of the species under consideration, i.e., the human kind. In Ref [9]., data show

that the population growth rate has been slowly decreasing at a rate *γ* of 0.017% per year, and is projected to doing so in the next decades.

For such value of *γ,* we estimate the time evolution of *r(t)* as

(5) $$\frac{dr}{dt}=-\gamma r\left(t\right),$$

giving an exponential behavior for *r(t)*. Note that if, in dimensionless unit, *γ* ~ 1, we end up into the linear case *r(t) ≈ r0 + γt*. In other words, the exponential case, for *γ* ~ 1, includes the constant rate case, i.e., the linear case. Adding Eq. (5) to Eqs. (1) and (2) we can now evolve the interaction human-forest with the addition of a time-dependent population growth-rate *r(t)*. Running in parallel the (stochastic) evolution of the technological level *T(t)* given by Eq. (4), we can estimate if the growth of *T(t)* hits the Dyson-sphere limit before the human-forest system reaches the no-return point, after which a catastrophic population collapse is inevitable (see [1] for details). Averaging over the stochastic realizations, we can estimate the probability of this event occurring.

**Figure 1.**
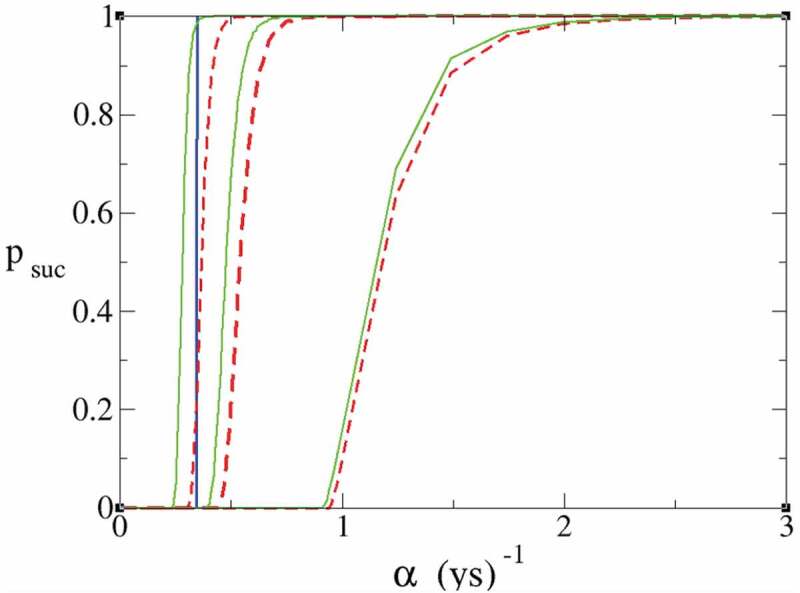
Probability of surviving without a catastrophic population collapse. Red dashed lines are obtained with constant *r* = 0.01 yr^−1^. Green lines are obtained with a time-dependent decreasing value of r(t). From right to left the values of *β* for the three couples of curves are *β* = 170, 300, 700. The blue line indicates the Moore’s law value *α* = 0.345 yr^−1^

We consider several scenarios depending on the value of the parameter *β* and the time dependence of *r*, with *β* in the range 170 < *β* < 700 and assuming an exponential decrease for the rate *r(t)*. We obtain that, for more realistic values of *β* < 600, the probability of survival remains very low even if it is slightly increased compared to the case of constant growth-rate *r*, confirming results obtained in [1]. For *β* = 700 though, we observe a dramatic increase from 10 − 20% (where the red dashed curve intersects the blue line in Figure 1) to more than 90% probability of avoiding collapse as compared to the case with constant *r*. Therefore, we find that only in the remote scenario where we were able to increase the carrying capacity to values of *β* > 600 and the technological growth-rate to the (rather optimistic) values of α∼ x> 0.3 yr^−1^, then a decreasing population growth-rate rate *r*, as stemming from recent data, would give a more positive outlook on our chances to avoid a catastrophic population collapse.
